# Analysis of 18 complex diffuse arteriovenous malformation cases treated with percutaneous radiofrequency ablation

**DOI:** 10.1186/s12872-021-02169-1

**Published:** 2021-08-03

**Authors:** Chun-Xiao Ge, Mao-Zhong Tai, Tao Chen, Ke-Lei Li, Zhen-Guo Xu, Zhong-Ping Qin

**Affiliations:** Special Department of Vascular Anomalies, Linyi Cancer Hospital , No. 6 of Lingyuandong Street , Linyi, 276001 Shandong Province China

**Keywords:** Arteriovenous malformation, Ethanol, Surgery, Complication, Radio frequency ablation

## Abstract

**Background:**

The aim of the present study is to evaluate the short-term efficacy and feasibility of radiofrequency ablation in the treatment of complex diffuse arteriovenous (AV) malformations.

**Methods:**

The data of 18 patients (8 male and 10 female) with complex AV malformations treated between December 2014 and June 2019 were analyzed retrospectively. The lesion area was 10 × 7 cm ~ 28 × 30 cm. Under duplex ultrasound guidance, the site with the most abundant blood flow signals in the lesion was percutaneously punctured with the radiofrequency ablation needle (electrode). The impedance automatic adjustment mode was adopted, and ablation was monitored usingduplex ultrasoundduring the entire process.

**Results:**

Of the included patients, 1 had a high fever after two rounds of treatment, 2 had transient hemoglobinuria, and 1 had tissue necrosis in the original ruptured tumor area as well as a penetrating defect in the cheek, which was repaired with a pedicled trapezius myocutaneous flap. In 9 patients who experienced bleeding, the bleeding stopped after one round of treatment. During the follow-up period of 1–5 years, there were 0 grade I (poor) cases, 0 grade II (medium) cases, 7 grade III (good) cases, and 11 grade IV (excellent) cases.

**Conclusion:**

The “high power and continuous” radiofrequency ablation technique conducted under real-time duplex ultrasoundmonitoring can completely destroy the deep core lesions of AV malformations and effectively control life-threatening massive hemorrhage; it is an effective alternative treatment method for complex diffuse AV malformations in which interventional embolization, sclerotherapy, and surgery are ineffective.

## Background

Arteriovenous (AV) malformation is a kind of congenital high velocity vascular malformation [1]. Intracranial AV malformations are reported frequently [2–5], but extracranial AV malformations are relatively rare [6]. They often occur in the maxillofacial neck [7], torso, and extremities [8, 9]. AV malformations appear during embryonic vascular development; most of the cases showed local red skin lesions after birth, with a slightly higher skin temperature, and several cases showed no obvious abnormal changes after birth. With the slow increase in age, lesions can develop; this development can be rapid during puberty or after trauma, and its treatment is recognized as a thorny problem in the industry. At present, there is no effective treatment available. Complete one-stage surgical resection is generally considered a highly thorough treatment. However, in some cases, the lesions are diffuse, the boundary is unclear, with lesions often invading deep important structures or organs, and the blood supply is so abundant that the lesions cannot be clinically resected. Other commonly used treatment methods are embolization and sclerosing agent injection [10], which have a good effect in the treatment of limited lesions but a poor effect in the treatment of diffuse and complex cases. Even if the lesions can be temporarily reduced and alleviated, it is easy to relapse in a short time [11]. Some patients who have undergone inappropriate treatment (including unilateral blood supply artery ligation, blood supply artery embolization, and partial lesion resection) are more likely to experience disease progression [12] and complex AV malformations, making the treatment more difficult.

Recently, radiofrequency ablation has mostly been used in the minimally invasive treatment of malignant tumors or solid lesions [13], which can effectively ablate the diseased vessels and tumors [14]; however, this method has rarely been used in the clinical treatment of vascular diseases, especially AV malformations. Between December 2014 and June 2019, 18 intractable complex cases were treated with percutaneous radiofrequency ablation under full-time duplex ultrasoundmonitoring in the Linyi Cancer Hospital; the curative effect was satisfactory. The report is presented in the text below.

## Methods

### Subjects

The data of 18 patients (8 male and 10 female) with complex AV malformations who were treated using percutaneous radiofrequency ablation in the Linyi Cancer Hospital between December 2014 and June 2019 were analyzed retrospectively. The total 18 cases are all extratruncular and infiltrating type based on the Hamburg Classification [15, 16]. The patients were aged 10–48 years, with a median age of 25.5 years. Among the cases, 14 had lesions in the maxillofacial region, 1 had lesions in the trunk, and 3 had lesions in the lower limbs; all of these lesions were diffuse, and most of them involved full-layer penetration from skin to mucosa. The lesion area of 10 × 7 cm ~ 28 × 32 cm was not good or ineffective before repeated embolization, sclerotherapy, or operation, and the lesions continued to develop; this included 6 cases of intraoral bleeding and 1 case of nasal bleeding.

### Imaging examination

All patients were examined via routine computed tomography angiography (CTA), which showed high-density shadow in the lesion area, obvious lesion enhancement, and unclear lesion boundaries. Vascular reconstruction showed a large number of tortuous and dilated thickened vascular masses and thickened blood supply arteries, along with a higher number of branches and abnormally large reflux veins on the affected sidecompared with the unaffected areas. A total of 15 cases were examined via feeding artery arteriography through femoral artery intubation under digital subtraction angiography (DSA).

### Treatment instruments and monitoring equipment

The radiofrequency ablation (CC-1–220 radiofrequency ablation system, ValleyLab Inc., USA) was used in the present study. The radiofrequency ablation system adopted the ValleyLab cool-tip TM RF ablation system, special cold circulation pump, cool-tip radiofrequency ablation needle (electrode), needle tip exposed 2 cm, and a matching special skin electrode. The ultrasonic instrument adopted the PHILLIPS HD15 color ultrasonic diagnostic instrument with an ultrasonic probe frequency of 2–5 MHz.

### Preparation before treatment

The routine hematological examination included blood routine, liver function, renal function, blood glucose, blood coagulation, D-dimer, positive and lateral chest X-ray, and ECG examination. The treatment plan and technology of the present study were demonstrated and approved by the Linyi Cancer Hospital ethics committee, and the patients or their parents signed a treatment-related written informed consent form. The ablation plan was created via multidisciplinary consultation.

### Treatment

The lesion size, range, depth, needle insertion direction, and blood flow were detected via intraoperative duplex ultrasound. Under duplex ultrasound guidance, the site with the most abundant blood flow signals in the lesion was percutaneously punctured with the radiofrequency ablation needle (electrode), the impedance automatic adjustment mode was adopted, and the ablation power was maintained at 60–130 W. During the ablation process, the lesion was vaporized using high signal intensity under real-time Duplex Doppler ultrasound monitoring. With the continuous lesion vaporization around the tip in the process, the tissue resistance increased, and the ablation instrument automatically stopped working; this kept the puncture needle fixed during the time. After the gas in the ablation lesion tissue dissolved, the human tissue resistance decreased, and the ablation equipment started to work automatically until the ablated diameter reached 2–3 cm and the ablation vaporization tissue became round. Then, the radiofrequency needle was slightly withdrawn and radially adjusted along the original puncture needle eye to the peripheral lesion area with a rich blood supply; this continued until the electrode tip and ablation vaporization area edge were 1 cm apart. The area with a diameter of approximately 2–3 cm was then ablated again and repeatedly adjusted and ablated in the same needle path until an area with a diameter of approximately 6 cm was formed. The ablation needle was then removed, and other lesions with the most abundant blood supply were selected for ablation treatment once again.

The ablation technique and treatment parameters changed according to the different site operation; the ablation power was 60–130 W in the maxillofacial region, 90–130 W in the deep lesion center, and 60–80 W in superficial or relative marginal lesions. The treatment time of each ablation was 60–90 min, with an average of 76 min. The next treatment was repeated in about 3 months in accordance with the imaging results.

*Postoperative care* No local compression or antibiotic application was required. After surgery, butanitol (Ketorlac Trimethanine Injection, Shandong New Era Co., Ltd., China) was given intravenously for 3 days. Lower extremity patients were given a low-molecular-weight heparin (Nadroparin Calcium injection, Glaxo Wellcome Production, France) subcutaneous injection. A total of 7 consecutive days were required for deep vein thrombosis prevention, maxillofacial patients do not.

### Observation and follow-up

After the operation, the patient body temperature was monitored, skin color at the puncture point was observed, skin or tissue necrosis was recorded, urine color and volume were paid attention to, and peripheral nerve motor and sensory function were evaluated. The CTA was reexamined every 3 months after discharge.

### Therapeutic effect evaluation

The long-term evaluation of the lesions was based on objective imaging indicators, and the therapeutic effect was evaluated with reference to the grading criteria put forward by Achauer et al. [17]: grade I (poor), with a lesion reduction of 0–25%; grade II (medium), with a lesion reduction of 26–50%; grade III (good), with a lesion reduction of 51–75%; and grade IV (excellent), with a lesion shrinkage of 76–100%.

### Statistical analysis

The Statistical Package for Social Sciences 20.0 program (IBM, Chicago, USA) was used to conduct the statistical analysis. The continuous variables of normal distribution were expressed as the mean ± standard deviation, the continuous variables of non-normal distribution were expressed as the median (interquartile range), and the categorical variables were expressed as frequency (percentage [%]). A *p* value of < 0.05 was considered statistically significant.

## Results

### General characteristics

The participant characteristics are listed in Table 1. The length of hospital stay was 7–13 days, with a median of 9 days. The patients were treated once every 3 months at a total of 3–6 times, with a median of 5 times.

**Table 1:** The characteristics of participants

Number	Sex	Age (years)	Lesion location of AVM	Tumor size (cm)	Symptoms	Treatment times	Efficacy
1	Female	24	Left breast of the back	28 * 32	Pain, palpitation, involvement of thoracic vertebrae and ribs	6	Grade III (good)
2	Female	21	Right side of the face and the base of the tong	18 * 12	Introral bleeding, ulceration	6	Grade IV (excellent)
3	Female	32	Right side of the face	20 * 15	Introral bleeding, ulceration, pain	5	Grade III (good)
4	Male	40	Left ear	23 * 20	Introral bleeding, ulceration	5	Grade IV (excellent)
5	Female	21	Left side of the face	20 * 16	Introral bleeding, ulceration	6	Grade IV (excellent)
6	Female	10	Right side of the face and ear	15 * 8	Continued enlargement of the lesion	4	Grade IV (excellent)
7	Male	26	Nose denomination	10 * 7	Nose bleeding	5	Grade III (good)
8	Female	10	Right side of the face	16 * 15	Continued enlargement of the lesion	4	Grade III (good)
9	Male	28	Tongue base and chin	15 * 12	Tongue bleeding	5	Grade IV (excellent)
10	Female	12	Right side of the face	11 * 8	Pain	5	Grade IV (excellent)
11	Female	45	Right side of the face	10 * 7	Introral bleeding, pain	4	Grade IV (excellent)
12	Female	28	Right feet	15 * 10	Ulceration, pain	3	Grade III (good)
13	Male	37	LEFT eyelid	21 * 16	Ulceration, pain	6	Grade IV (excellent)
14	Female	29	Right feet	11 * 7	Continued enlargement of the lesion, ulceration	4	Grade IV (excellent)
15	Male	15	Right side of the face	17 * 14	Continued enlargement of the lesion	5	Grade III (good)
16	Male	43	Left upper lip	10 * 8.5	Introral bleeding	4	Grade IV (excellent)
17	Male	25	Left side of the face	13 * 10	Introral bleeding,	4	Grade III (good)
18	Male	19	Left feet	10 * 8	Pain	3	Grade IV (excellent)

### Adverse reactions

A total of 9 cases presented with fever and recovered after symptomatic treatment. Transient hemoglobinuria occurred in 4 patients after operation and intravenous infusion of 5% sodium bicarbonate solution alkalized urine, and tissue necrosis in the original ruptured tumor area, along with a penetrating defect in the cheek, occurred in 1 case patient; this defect was repaired with a pedicled trapezius myocutaneous flap and healed. A total of 2 patients experienced postoperative numbness with no dyskinesia and recovered 3 months after neurological treatment.

### Curative effect evaluation

There were 9 cases of intraoral hemorrhage and 1 case of nasal hemorrhage. After one ablation treatment, the bleeding stopped, and no more bleeding occurred. During the follow-up of 1–5 years, there were 0 grade I (poor) cases, 0 grade II (medium) cases, 7 grade III (good) cases, and 11 grade IV (excellent) cases.

### Typical cases

**Fig. 1:**
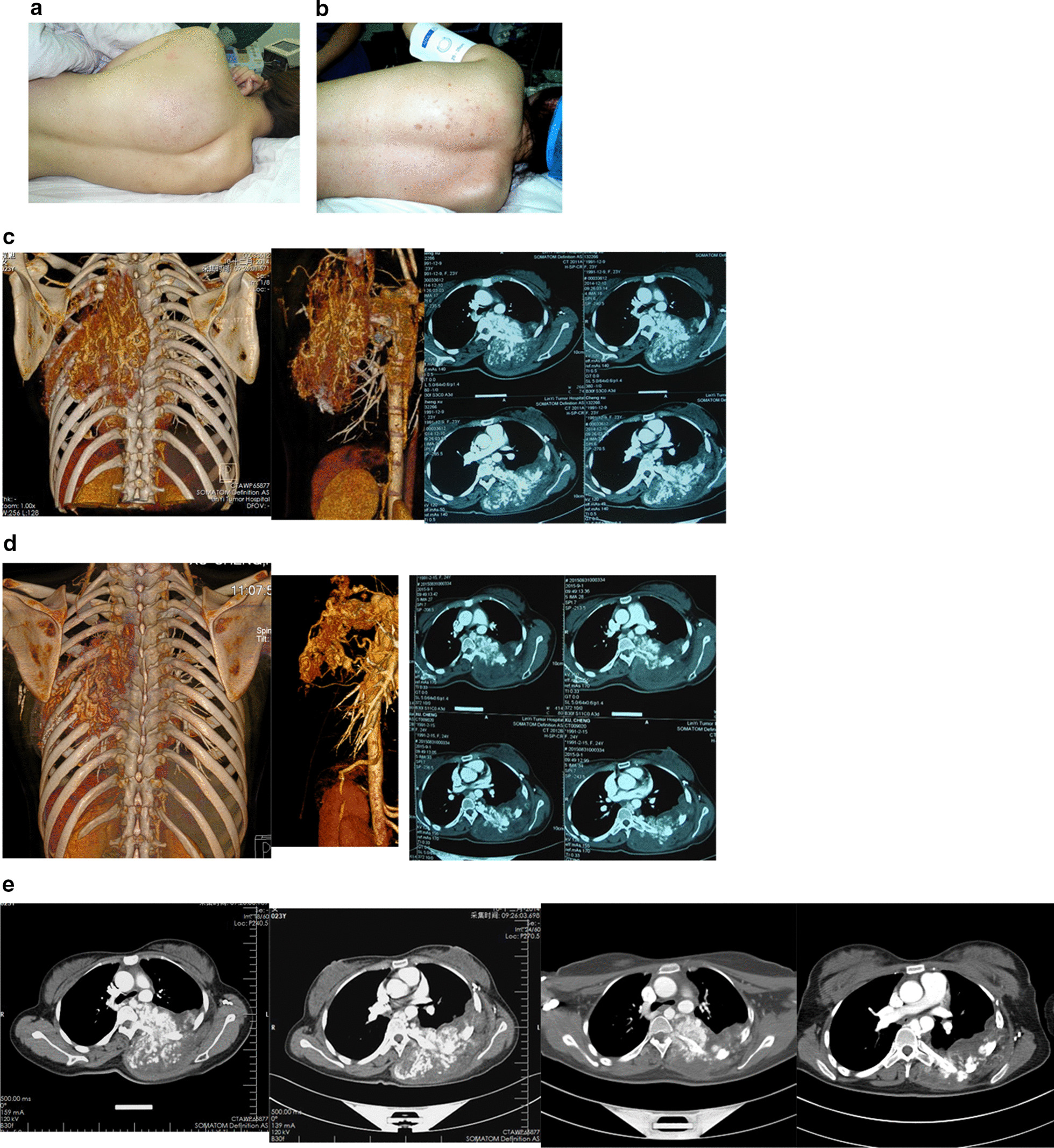
The comparison of diseased blood vessels and collateral vessels before and after treatment. **a** Before treatment, the lesion of the left chest and back was obviously raised and convex. **b** After treatment, the lesion of the left chest and back was obviously reduced, and multiple ablation needle marks were seen on the surface skin. **c** The pre-treatment CTA results showed that the lesion involved the left posterior chest wall, bone destruction, and multiple ribs and thoracic vertebrae. The lesion was obviously protruded into the chest, multiple blood supply arteries were enlarged, directly from the thoracic aorta, the collateral vessels were extremely abundant, and the lesions were obviously enhanced. **d** After treatment, the left erector spine lesions and intercostal muscles present in the CTA were significantly reduced and thinned. Most of the original enhanced lesions showed a low density, and the blood supply and collateral vessel number decreased significantly. **e** The CTA case comparison before and after treatment (3-year reexamination) showed that the left erector spine lesions and intercostal muscles had been further reduced and thinned. Most of the original enhanced lesions showed a low density, and the blood supply arteries and collateral vessels decreased significantly

**Fig. 2:**
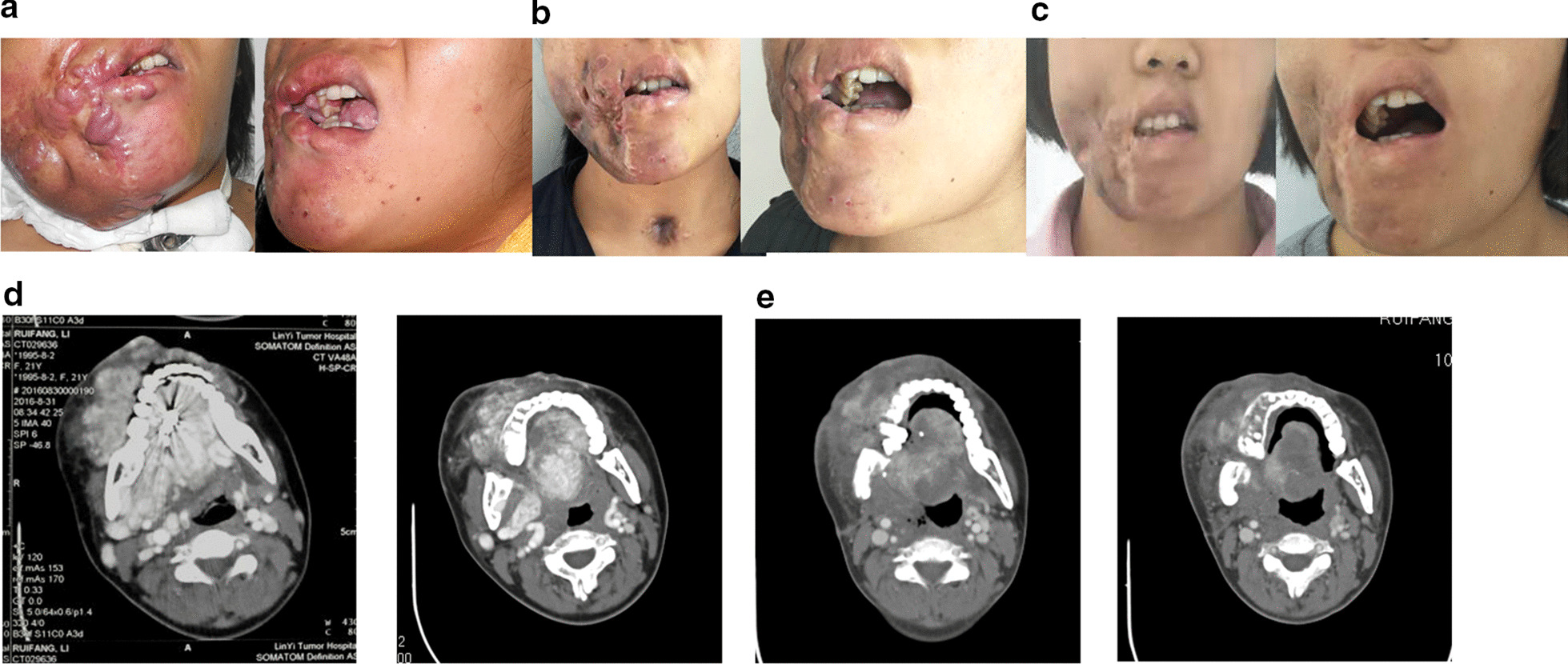
Facial lesion comparison before and after treatment. **a** Appearance before treatment: facial lesions have a high skin temperature, with obvious arterial pulsation, intraoral gingival thickening, tongue body thickening, and tongue body thickening. **b** After treatment, the facial lesions significantly reduced, the color recovered, the tongue body became smaller, and the gingival lesions disappeared. **c** After 2 years of treatment, the face was basically symmetrical, the skin color was close to normal, and there was no facial paralysis. **d** The CTA showed diffuse enhancement of right maxillofacial tongue lesions, and right neck A significantly circuitous thickening and increased. The diseased tongue filled the whole mouth. The pharyngeal cavity narrowed. **e** After treatment, the CTA showed low density changes in over 85% of the original tongue floor and facial enhancement lesions. Most of the abnormal blood supply arteries and collateral vessels disappeared, the diseased tongue body was significantly reduced, and the pharyngeal cavity was recovered

The first typical case was a 23-year-old female from the Jiangsu Province who had extensive AV malformations in the left chest wall. She had previously undergone 5 rounds of superselective arterial embolization and multiple injection sclerotherapy outside the hospital; however, the effects of these treatment methods were not good. The lesion continued to grow, and the pain in the lesion area was obvious in the past year. The pain was severe at night, affecting sleep and causing palpitation, shortness of breath, and difficulty getting up the stairs. The patient was admitted to the hospital on December 1, 2014. A physical examination showed an obvious tumor protuberance on the left side of the chest and back, an obvious increase in local skin temperature, palpable lifting pulsation and tremor, and a grade IV wind-like murmur during auscultation. Diagnosis: extensive AV malformations in the left chest wall with cardiac insufficiency. Multidisciplinary consultation showed that the patient had a wide range of lesions involving major thoracic vessels and other important structures, bone destruction, and multiple ribs and thoracic vertebrae (see Fig. 1). The surgical approach was difficult, and the possibility of causing uncontrollable massive bleeding during the operation was high. The risk of this method is still great even if the tissue defect is difficult to repair after resection; thus, it was not a suitable choice for the operation. Additionally, many thick blood supply arteries of the lesion originated directly from the thoracic aorta, and the collateral vessels and AV fistulas were extremely abundant (see Figs. 1, 2). Arterial and local interventional embolization may not be effective; however, embolization has been carried out several times in many large hospitals at a higher level. Clinical practice has proven that the lesion is still progressive and uncontrolled after treatment, and percutaneous radiofrequency ablation under ultra-real-time color monitoring can be performed. After 4 rounds (3–4 points each) of radiofrequency ablation treatment, the lesion was significantly reduced and the localized tenderness completely relieved. Moreover, the core tumor disappeared, the cardiac function gradually recovered, and shortness of breath and palpitations improved. Reexamination 3 years after treatment: the lesions of the left erector spine and intercostal muscles were further reduced and thinned; over 75% of the original enhanced lesions showed low density changes. The patient had a normal life with a grade III (good) curative effect evaluation.

**Fig. 3:**
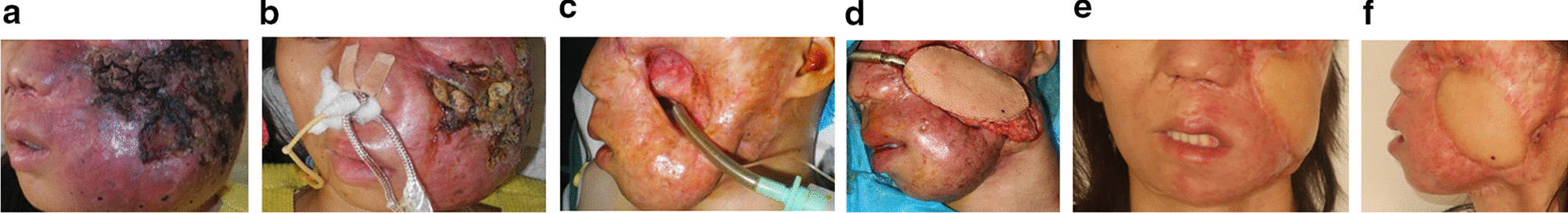
Clinical efficacy after treatment. **a** Before treatment, the disease was complicated with a huge ulcer, and the treatment of multiple interventional embolization was ineffective. **b** Postnasal compression using a water-filled Foley catheter was conducted to stop severe nasal bleeding. **C **After one round of radiofrequency ablation, the bleeding in the nasal cavity stopped. All lesions disappeared after four rounds of radiofrequency ablation. **d** The trapezius myocutaneous flap was used to repair the facial defects. **e** After 30 months of treatment, no recurrence was found. **f** After 30 months of treatment, no recurrence was found

The second typical case was a 21-year-old female with extensive AV malformations in the floor of the right tongue. The patient had undergone embolization and sclerotic injection many times in several major hospitals; however, the effects were poor, and the patient’s condition had gradually become aggravated. Following the sudden occurrence of oral bleeding and compression hemostasis, the patient was transferred to Linyi Cancer Hospital for treatment. The day after admission, the patient presented with jet bleeding, and the hemostatic effect of compression was not good. Tracheotomy was performed urgently, and iodoform gauze was sutured at the buccal mucosa of the affected side to stop the bleeding. Further examination of the CTA and DSA showed typical AV malformations of the right maxillofacial mouth and tongue as well as involvement of the maxilla and mandible (see Fig. 3a–f). Resection of the right mandible and multiple embolization of soft tissue lesions were performed under general anesthesia. However, the lesions continued to develop and were not controlled, especially those in the tongue and the floor of the mouth; this made it difficult for the patient to breathe and eat. Simultaneously, many instances of mucosal erosion and bleeding occurred in the oral lesion area after the operation. Following multidisciplinary consultation, percutaneous and mucosal radiofrequency ablation was performed under general anesthesia. After 1 round of ablation, there was no intraoral bleeding, and after 3 rounds (3–4 points each) of radiofrequency ablation, the tongue, mouth, and maxillofacial region lesions were reduced by over 85%. Furthermore, the core main tumor disappeared, the tracheal cannula and gastric tube were removed, and the patient’s breathing and eating returned to normal. The curative effect was evaluated as grade IV (excellent).

## Discussion

AV malformation is a vascular disease with a high flow velocity, which has the greatest influence on patient tissue structure and function [18]. The core tumor nidus in the lesion comprised a large number of micro-AV fistulas. This special micro-AV shunt reduced the resistance of blood flow in the lesion area; this was equivalent to a pump-like structure. A large amount of blood did not pass through the capillary bed and continuously flowed back to the vein through the short circuit structure. The relatively low resistance and ischemic state of the capillary bed were two important features of hemodynamic AV malformation changes. With the progress of the disease, there may be thickening of arteries and veins, involvement of surrounding tissues, lack of capillary bed structure (in the later disease stage), tissue ischemia, ulceration and nonunion, massive hemorrhage, etc.; therefore, the clinical treatment is very difficult. It is also very important to recognize the AV shunts early, as their presence could place the patients into a class of highly endangered individuals in regard to recurrence risk and complications.

Endovascular embolization was previously the most commonly used treatment for vascular malformation. Occlusion of the feeding arteries and the abnormal vascular nests should be the main objectives of treatment; surgical removal or reduction of the hemodynamic AV malformations effects is the second option. Sclerotherapy, in which ethanol is injected through a catheter or via direct puncture, is another widely accepted treatment method. Laser therapy is not often used for the treatment of AV malformations; however, in some cases, ultrasound-guided percutaneous laser therapy may be effective in the treatment of smaller localized defects.

The principle AV malformation treatment should eliminate micro-AV shunts and improve the tissue blood supply, to which this elimination is essential. It was generally believed that, if possible, complete surgical resection is the best treatment method option. Surgical resection is mostly suitable for relatively limited or superficial lesions where the lesion is wide and deep, involving important nerves and vessels. However, the operation can easily cause fatal intraoperative bleeding, and the defect is difficult to repair after resection. Inappropriate conduction of partial surgical resection could lead to a rapid lesion progression, such as ischemia, bleeding, ulcers, and other changes [19]. If combined with deep bone tissue lesions, the disease may become more intractable. Similarly, simple embolization or sclerotherapy is mostly ineffective in the treatment of diffuse lesions [20]. Even with the use of the strongest anhydrous ethanol available, diffuse lesions can only be temporarily improved or relieved. Moreover, their progress is difficult to control. It was generally advocated that when combined with surgical treatment, these methods can achieve a better effect with a relatively low recurrence rate [11]. It has been reported that coil embolization can only be used for larger AV shunts and that a large number of residual AV shunts after treatment will accelerate the disease onset and lead to disease progression [21]. Fluid embolization can achieve more AV fistula embolization, transarterial, venous, and percutaneous embolization [22]. However, the 18 cases included in the present study were previously treated with multiple embolization or surgery in many hospitals; the curative effects were not good or ineffective, and the lesions continued to progress. Several cases were complicated with massive tumor ejection hemorrhage and were rushed to Linyi Cancer Hospital for further treatment after compression and hemostasis.

Complex AV malformations, usually including cases unresponsive to many kinds of treatment in the early stage or cases with lesions that continued to progress after treatment, are often encountered in clinical practice. All 18 cases in the present study were consistent with the above-written conditions. Furthermore, 9 cases had intraoral or nasal hemorrhage, 7 cases had deep skeletal lesions, and 9 cases had extensive diffuse lesions with penetrating skin and mucosa involvement and a deep base involving important structures. In the above-mentioned complex cases, there was an urgent need to find an effective alternative treatment.

Percutaneous radiofrequency ablation is an interventional therapy that has been rising in recent years. Its basic principle is that under the excitation of a high-frequency-alternating current, the charged particles in the tissue around the electrode produce high-speed concussion and friction heat. Next, the secondary tumor tissue cells and interstitial blood vessels are dehydrated, leading to protein degeneration, degeneration, and necrosis. The lesions then gradually atrophy and disappear through immune phagocytosis. At present, this method is primarily used in malignant liver tumors, malignant lung tumors, hepatic hemangiomas, thyroid tumors, etc. [23]; however, its use has rarely been reported in the treatment of vascular diseases, with the exception of hepatic hemangiomas [24]. Malignant tumors have an abundant blood supply and large number of AV fistula structures, making it easy to relapse or even accelerate the treatment progress. In recent years, ablation therapy has been used in the clinical treatment of a variety of malignant tumors, and the method has achieved good results [25]. Inspired by these findings, we applied this technique to cases of complex AV malformations in which other methods had failed many times; the process was conducted in December 2014 after multidisciplinary consultation. In the first typical case, the patient had previously undergone embolization and sclerotherapy several times in a number of hospitals at a higher level. However, the disease continued to progress and became complicated with cardiac insufficiency. Under real-time, multi-site percutaneous duplex ultrasound monitoring, a high-power continuous ablation in the core lesion area was performed. After 1 round of treatment, it was found that the lesion in the ablation area had significantly reduced and that the original lesion had been obviously enhanced with a low density. After 3 rounds of treatment, the pathological changes reduced significantly, and the main tumor disappeared. Moreover, local pain was completely alleviated, shortness of breath and palpitations gradually improved, and cardiac function recovered.

A different from of the “low power, dynamic” ablation technique was used for the treatment of venous malformations, and the “high power and continuous” ablation technique was used for the treatment of AV malformations [26]. AV malformations have an abundant blood supply, which can take away part of the heat generated by ablation; this can cause effective damage to a large number of AV fistulas. Under color Doppler ultrasound guidance, the core tumor with the most abundant blood flow signals can be accurately punctured for ablation; however, caution is required in order to not penetrate or ablate the lesion excessively. If this happens, the disease can be complicated with fatal intracavitary hemorrhage. Usually, the more abundant the blood supply, the larger the lesion area and the higher the power requirement. In the present study, the ablation power was 90–130 W in the central areas of deep lesions and 60–80 W in superficial or relatively marginal lesions [27]. After ablation, there were respective reductions in core lesion range, blood supply, and power. All patients were treated with staged ablation. The lesions were reduced to varying degrees, the bleeding stopped, and the ulcers healed. The outcomes of the present study revealed that staged ablation (with an interval of approximately 3 months) effectively blocked the tumor center blood supply, effectively controlling terrible fatal bleeding in time. The lesion progress was not accelerated due to residual lesions. These findings provide a new method for the control of AV malformation complicated with bleeding.

In addition to tissue necrosis in the ablation area, other radiofrequency ablation complications included postoperative fever and transient hemoglobinuria. A total of 9 patients developed postoperative fever, which was related to postoperative heat absorption, and returned to a normal state after treatment with glucocorticoid. All the follow-up cases were treated with an intravenous infusion of glucocorticoid, and there were no more cases of fever. A total of 4 patients developed transient hemoglobinurias, which disappeared after intravenous infusion of 5% sodium bicarbonate solution alkalized urine. Childs et al. [24] reported that ablation of lesions adjacent to the nerve pathway area can cause nerve injury, including direct thermal injury and postoperative secondary nerve edema. In the present study, there were 5 cases of maxillofacial AV malformations surrounding the facial nerve trunk or main branches; of these, 4 cases were complicated with facial nerve dysfunction and facial paralysis when treated in other hospitals. Otherwise, the core lesions could not be eliminated, and the curative effect was not good. In the other 4 cases, the maxillofacial lesions were mainly located in the floor of mouth, tongue, lip, and mental face. The facial nerves were preserved, and there was no facial paralysis. A total of 2 patients experienced postoperative numbness with no movement disorder, and recovered 3 months after symptomatic treatment (e.g., nerve management and nourishment).

Radiofrequency was previously introduced to close varicose veins. In varicose veins, thermoablation is the routine treatment procedure selected by phlebologists. However, varicose vein thermoablation can only alleviate patients' clinical symptoms; it cannot cure the disease. At present, radiofrequency ablation is an advanced, minimally invasive method that may be valuable for varicose vein treatment. Recently, phlebologists have begun performing radiofrequency worldwide in the treatment of vascular malformations.

*Study Limitations* The present study has several limitations: (1) the present study is not a randomized controlled trial; (2) the study is a single-center trial (a future multiple-center trial is still required); and (3) the sample size is strongly limited (a future larger trial with more participants is necessary).

## Conclusion

Radiofrequency ablation is effective in the treatment of complex intractable AV malformations. This provided a new idea for patient treatment. It was suggested that the initial implementation should be carried out after multidisciplinary consultation. The joint participation of interventional physicians and ultrasound physicians with experience in tumor ablation treatment is still required. Accurate positioning, puncturing, and ablation skills were the premise of ensuring the curative effect and the key to reducing complication occurrence.

## Data Availability

All data generated or analyzed during this study are included in this published article.

## Abbreviations

AV: Arteriovenous
CTA: Computed tomography angiography
DSA: Digital subtraction angiography

## References

[1] Shah K, Srinivasan B, Ethunandan M, Pratt C (2017). Arteriovenous malformation of the jaws: a black hole for the GDP—a case report. Dent Update.

[2] Cenzato M, Dones F, Marcati E, Debernardi A, Scerrati A, Piparo M (2017). Use of laser in arteriovenous malformation surgery. World Neurosurg.

[3] Al-Smadi A, Shokuhfar T, Johnston A, Alden TD, Bowman R, Shaibani A (2017). The rare case of a large complex intraosseous cranial arteriovenous malformation with successful multidisciplinary management. J Neurosurg Pediatr.

[4] Osada Y, Endo H, Sato K, Matsumoto Y, Matsumoto Y, Endo T, Fujimura M (2017). Successful presurgical endovascular management of venous sinus thrombosis associated with high-grade cerebral arteriovenous malformation: a case report. Interv Neuroradiol.

[5] Park CK, Choi SK, Lee SH, Choi MK, Lim YJ (2017). Clinical outcomes and radiosurgical considerations for pediatric arteriovenous malformation: influence of clinical features on obliteration rate. Childs Nerv Syst.

[6] Kumar A, Mittal M, Srivastava D, Jaetli V, Chaudhary S (2017). Arteriovenous malformation of face. Contemp Clin Dent.

[7] Neeta S, Rao R, Upadya M, Keerthi P (2017). Arteriovenous malformation of face: a challenge to anesthesiologists. Anesth Essays Res.

[8] Ramírez-Senent B, Abadal JM, Vázquez E, Lago I, Gálvez E, Araujo MA (2017). Endovascular management of a giant high-flow lower limb arteriovenous malformation. Vasc Endovasc Surg.

[9] Diep J, Dandu K, Xiong M, Shulman SM, Gonzalez-Fiol AJ (2017). Airway arteriovenous malformation in pregnancy. Can J Anaesth.

[10] Li J, Liu H, Ye L (2015). Coil embolization for a vast and complex arteriovenous malformation in the posterior mediastinum. Int J Clin Exp Med.

[11] Liu AS, Mulliken JB, Zurakowski D, Fishman SJ, Greene AK (2010). Extracranial arteriovenous malformations: natural progression and recurrence after treatment. Plast Reconstr Surg.

[12] Couto JA, Huang AY, Konczyk DJ, Goss JA, Fishman SJ, Mulliken JB (2017). Somatic MAP2K1 mutations are associated with extracranial arteriovenous malformation. Am J Hum Genet.

[13] Idiz UO, Aysan E, Elmas L, Yildiz S, Akbulut H (2017). The place of elastography in evaluating the efficacy of radiofrequency ablation of thyroid nodules. Am Surg.

[14] Adam LC, Murali N, Chapiro J, Geschwind JF (2017). Science to practice: molecular-targeted drug delivery in combination with radiofrequency ablation of liver cancer: a magic bullet?. Radiology.

[15] Carqueja IM, Sousa J, Mansilha A (2018). Vascular malformations: classification, diagnosis and treatment. Int Angiol.

[16] Bastide G, Lefebvre D, Jaeger JF (1990). The organogenesis and anatomy of vascular malformation. Int Angiol.

[17] Achauer BM, Chang CJ, Vander Kam VM (1997). Management of hemangioma of infancy: review of 245 patients. Plast Reconstr Surg.

[18] Kohout MP, Hansen M, Pribaz JJ, Mulliken JB (1998). Arteriovenous malformations of the head and neck: natural history and management. Plast Reconstr Surg.

[19] Hasnaoui N, Gérard E, Simon E, Guillet J (2017). Massive bleeding after a tooth extraction: diagnosis of unknown arteriovenous malformation of the mandible, a case report. Int J Surg Case Rep.

[20] Masiello R, Iadevaia C, Grella E, Tranfa C, Cerqua F, Rossi G (2015). A case of multiple unilateral pulmonary arteriovenous malformation relapse: efficacy of embolization treatment. Open Med (Wars).

[21] Funaki B, Funaki C (2016). Embolization of high-flow arteriovenous malformation. Semin Interv Radiol.

[22] Nassiri N, Crystal DT, Hoyt C, Shafritz R (2017). Chronic refractory venous ulcer exacerbated by a congenital pelvic arteriovenous malformation successfully treated by transarterial Onyx embolization. J Vasc Surg Venous Lymphat Disord.

[23] Andresen NS, Buatti JM, Tewfik HH, Pagedar NA, Anderson CM, Watkins JM (2017). Radioiodine ablation following thyroidectomy for differentiated thyroid cancer: literature review of utility, dose, and toxicity. Eur Thyroid J.

[24] Childs DD, Emory CL (2012). Successful treatment of intramuscular venous malformation with image-guided radiofrequency ablation. J Vasc Interv Radiol.

[25] Cirimbei C, Rotaru V, Chitoran E, Pavaleanu O, Cirimbei SE (2017). Immediate and long-term results of radiofrequency ablation for colorectal liver metastases. Anticancer Res.

[26] Brown DB, Brandes SB (2005). Radiofrequency ablation of a recanalized renal arteriovenous malformation. J Vasc Interv Radiol.

[27] Chen AL (2019). The relationship between ablation time, power and ablation range. Contemp Med.

